# Bioinformatic Identification of TP53 Gene Mutation Hotspots in Colorectal Cancer

**DOI:** 10.3390/ijms25126612

**Published:** 2024-06-15

**Authors:** Zsolt Kovács, Haruhiko Sugimura, Tamás Attila György, Eva Osvath, Felix Manirakiza, Simona Gurzu

**Affiliations:** 1 Department of Pathology, “George Emil Palade” University of Medicine, Pharmacy, Science and Technology of Targu Mures, 540139 Targu Mures, Romania; zsolt.kovacs@umfst.ro (Z.K.); gyorgytomims@gmail.com (T.A.G.); osvath_evi@yahoo.com (E.O.); 2 Research Center of Oncopathology and Translational Research (CCOMT), 540139 Targu Mures, Romania; 3 Sasaki Institute Sasaki Foundation, Tokyo 101-0062, Japan; hsugimur@po.kyoundo.jp; 4 Department of Oncology, Clinical County Hospital, 540140 Targu Mures, Romania; 5 Department of Pathology, School of Medicine and Pharmacy, College of Medicine and Health Sciences, University of Rwanda, Kigali P.O. Box 3286, Rwanda; manirafelix@gmail.com; 6 Romanian Academy of Medical Sciences, 030167 București, Romania

**Keywords:** TP53 gene, colorectal cancer, bioinformatics, mutation hotspots

## Abstract

Mutations and inactivation of the TP53 gene are frequently observed in various types of malignancies. Precise knowledge of the genetic structure and detection of mutation hotspots are crucial, as these indicate a high probability of developing cancer. The aim of our study was to perform the bioinformatic detection of mutation hotspots in the TP53 gene in patients diagnosed with malignant colon neoplasms using self-developed software (version 1). We compared TP53 gene sequences from 50 healthy individuals with those from 50 patients diagnosed with colorectal carcinoma. Of the 50 samples from cancer patients, the most frequent mutations were observed in exons 5 and 8 (12 mutations per exon) and gene sequences of 12 samples, which differed from those of the 50 samples from healthy individuals. Based on our results, the distribution of mutations in the TP53 gene structure was not even across different exons. By comparing the gene sequences of healthy individuals with those of colon cancer samples, we conclude that structural changes occurring in similar gene regions are not associated with increases in susceptibility to malignancies in every case, namely, that the pathological mechanism is multifactorial.

## 1. Introduction

Cancer is a multifaceted disease characterized by uncontrolled cell proliferation, which is often driven by genetic alterations that disrupt critical cellular processes. Mutations and inactivation of the TP53 gene can be observed in several malignancies. Although the direct mechanism is not yet fully understood, we can nevertheless clearly see that, in a physiologically healthy state, this gene plays a crucial role in providing protection for the body by preventing cancerous cell proliferation [1]. Accordingly, gaining precise knowledge of the genetic structures is important, as is screening for the detection of potential mutation hotspots, the presence of which could indicate an increased probability of developing cancer.

The role of the TP53 gene is essential in regulating DNA repair and cell division and is therefore often called the “guardian of the genome” [2]. It functions by coding for the p53 protein, which acts as a tumor suppressor. By regulating cell division, it prevents uncontrolled growth and proliferation in cells [1,3]. The p53 protein is in the cell nucleus and attaches directly to the polynucleotide chain of the DNA. If any carcinogenic agent produces harmful effects, the p53 protein plays an important role in governing how the cell’s life continues to unfold. If the damage inflicted on the DNA chain can be repaired, it will stimulate other repair genes through its gene activation function. On the other hand, if the damage suffered is irreparable, p53 activates the process of apoptosis. By preventing cells from containing damaged or mutated DNA, p53 plays an important part in preventing tumorigenicity. Most mutations occurring in the TP53 gene are missense mutations that lead to the synthesis of a p53 protein with a malformed structure that will be unable to fulfil its function, allowing the uncontrolled proliferation of cells carrying mutated and damaged DNA [4,5,6]. Inactivating mutations in the TP53 gene can lead to the accumulation of genetic errors and unrestrained cellular growth, thereby contributing to tumorigenesis and cancer progression.

Somatic mutations in the TP53 gene appear in approximately half of human malignancies, making it the most frequently mutated gene in various cancers. Mutations often occur in breast cancer, lung cancer, Li–Fraumeni syndrome, and colorectal carcinoma [7]. In the latter, the frequency of TP53 gene mutations is approximately 45–54% in sporadic colorectal cancer [4]. Consequently, this is one of the most common types of cancer in which somatic mutations of the TP53 gene can be observed, and several studies have shown that these TP53 mutations can be associated with an unfavorable prognosis and a high metastatic risk [8]. Consequently, a comprehensive understanding of the mutational landscape of the TP53 gene in this malignancy is crucial for improving early detection, risk stratification, and the development of targeted therapeutic strategies.

Although the significance of TP53 gene mutations in colorectal cancer is well established, the distribution and frequency of specific mutation hotspots within the gene remain incompletely characterized. Mutation hotspots are regions of genes that exhibit a higher propensity for alterations, potentially owing to increased vulnerability or exposure to mutagenic factors. Identifying these hotspots could provide valuable insights into the molecular mechanisms underlying colorectal cancer development and progression, as well as inform the design of diagnostic and therapeutic approaches tailored to the specific mutational profiles of individual patients.

Considering this, it would be important to adapt a procedure that may facilitate the structural analysis of the TP53 gene sequenced from samples of colon cancer patients based on already existing and known data. The bioinformatics approach provides a good alternative, enabling a comparison between the gene sequences of healthy samples and those of cancerous samples. Furthermore, bioinformatics analysis enables the identification of spots in which alterations from the normal genetic structure can be detected most frequently. Knowledge of these may not only serve as a reference point when analyzing further samples but also open up avenues for therapeutic approaches in modern medicine, such as gene therapy. Therefore, the aim of our study was to detect mutation hotspots in the TP53 gene in patients diagnosed with colon cancer using self-developed bioinformatics software (Version 1). Our program facilitated the detection and characterization of mutations, enabling a deeper understanding of the mutational landscape of the TP53 gene in this malignancy. The findings from this analysis could contribute to ongoing efforts in cancer research and clinical practice, ultimately leading to improved patient outcomes.

## 2. Results

**Figure 1:**
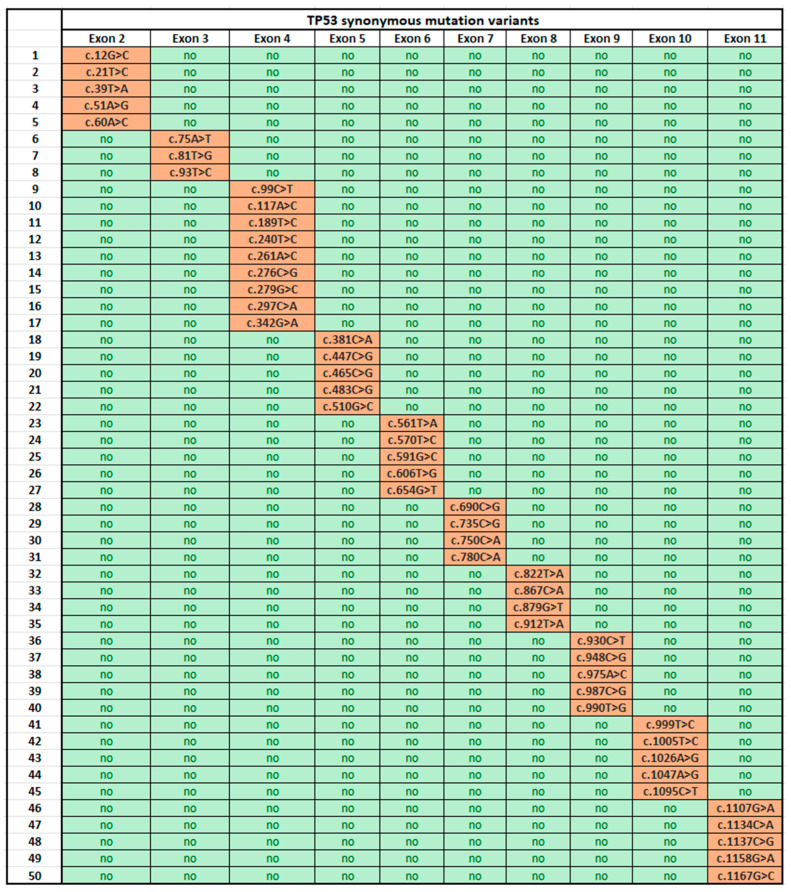
TP53 gene synonymous mutations in samples from healthy individuals. The exons without any described alterations are highlighted in green and are labeled “no”. Those carrying synonymous mutations are highlighted in orange.

**Figure 2:**
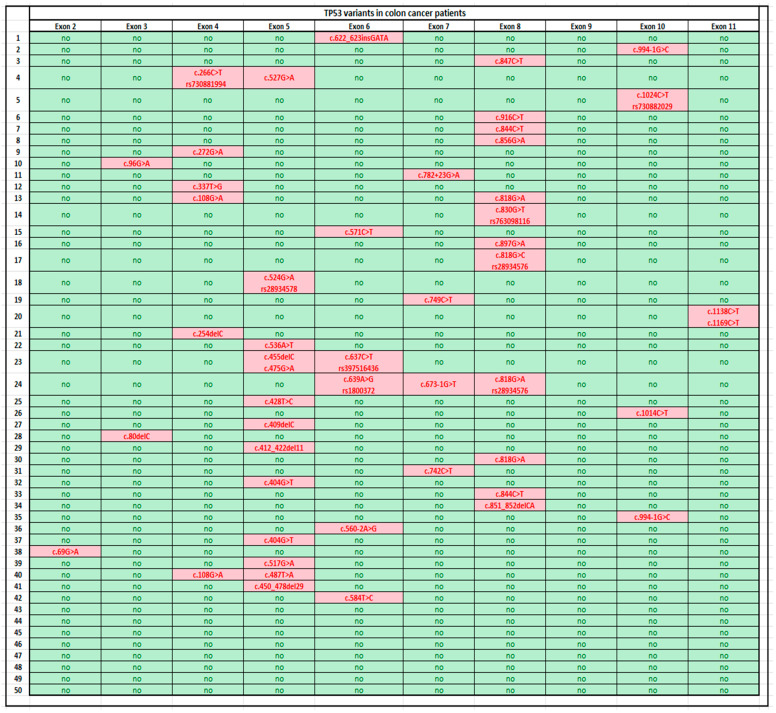
TP53 variants in samples from colon cancer patients. The exons in which the computer program failed to identify any alterations from the reference sequence are highlighted in green and are labeled “no”, while the exons containing the mutations by the computer program are highlighted in red, and each mutation was accompanied by a description of the type and position of the alteration.

For ease of reading, we sorted the samples into two groups, i.e., those of healthy individuals [9] and those with colon cancer, and the TP53 gene mutations in these two groups were presented in two respective tables that were separated and categorized by individual exons (Figure 1 and Figure 2).

We have described the exons containing synonymous mutations with their precise positions relative to the reference sequence NM_000546.6, and each one is presented in the table in the column corresponding to the altered exon.

Based on the results of the comparison, the sequences of the TP53 gene obtained from the samples of 50 patients with colon cancer showed alterations in the structure of at least one of the exons in the gene in 42 (84%) cases and no alteration in 8 (16%) cases. For the 42 samples with alterations, the most frequent mutations were observed in exons 5 and 8 (12 mutations for each exon), and 12 of the cancerous samples examined differed from the healthy samples. For exon 5, we were able to observe 12 mutations among the samples we examined; these were relatively spread between positions 400 and 500, and it is of note that of the 12 mutations, only 2 occurred in the same position 404. In the case of exon 8, these alterations were located primarily between positions 815 and 860; however, it is noteworthy that four out of twelve mutations appeared in position 818, while two occurred in position 844.

Regarding the number of mutations, exons 4 and 6 were the second most common, with alterations occurring in six cancerous samples for each exon. In exon 4, alterations were mostly observed between positions 250 and 300, but two of the cancerous samples each had a mutation present at position 108. For exon 6, we detected mutations primarily between positions 570 and 640; two cancerous samples showed alterations in positions that were adjacent, namely positions 637 and 639. Exons 7 and 10 had four mutations each. The mutations were mainly between positions 670 and 750 in exon 7, whereas for exon 10, they appeared between positions 990 and 1025; notably, there were two cases of alterations found in position 994. Among the remaining exons, alterations occurred once in exon 2 (position 69), twice in exon 3 (positions 80 and 96), and once in exon 11 (position 1169), respectively. The uneven distribution of mutations was further highlighted by the fact that we were unable to detect even a single alteration in exon 9 among the cancerous samples we analyzed.

**Figure 3:**
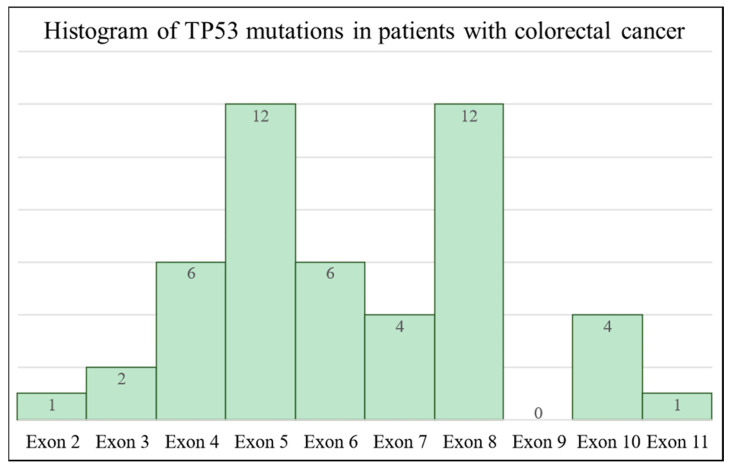
A histogram of TP53 gene mutations in patients with colorectal cancer. The distribution of mutations detected in the TP53 gene in patients with colorectal cancer is shown based on their positions in the exons of the gene.

Furthermore, a histogram of TP53 gene mutations in patients with colorectal cancer provides a good illustration of the exon-based distribution of mutations (Figure 3).

By examining the types of mutations that occurred in the exons examined, it can be observed that most of them correspond to substitution mutations being either single nucleotide polymorphisms or single nucleotide variants, but on a few occasions, we were able to observe deletions (e.g., c.80delC, c.254delC, and c.455delC) or, more rarely, insertions (e.g., c.622_623insGATA).

Based on our findings, it can be said that the distribution of mutations in the TP53 gene structure cannot be considered even, since for certain exons, such as exons 5 (alterations in 12 samples) and 8 (alterations in 12 samples), structural differences are far more frequent than those in other exons, such as exon 9, where we failed to detect even a single alteration among the cancerous samples analyzed. For the remainder of the exons, mutations were observed in roughly the same number of samples (exons 4 and 6 each had alterations in six samples, and exons 7 and 10 each had alterations in four samples, respectively), which led us to believe that in terms of the samples examined, mutation hotspots do indeed exist between positions 400 and 500 of exon 5, but mainly in position 404 (c.404G>T), as well as between positions 815 and 860 of exon 8, but mainly in loci 818 (c.818G>A and c.818G>C) and 844 (c.844C>T), where the structural changes could lead to increased predispositions for developing colorectal carcinoma. It can be hypothesized that the polynucleotide chain is more vulnerable and sensitive at these points, which might increase the likelihood of mutations appearing in these regions [10,11,12].

With regard to the samples deemed healthy, which contain synonymous mutations, it can be said that these mutations occur in a positional range similar to that of mutations identified in patients diagnosed with colon cancer, not in the same position but in positions proximal to those of the mutations occurring in colon cancer cases. In exon 5, we observed synonymous mutations among the samples deemed healthy at positions 447 (c.447C>G), 465 (c.465C>G), and 483 (c.483C>G), whereas in the samples of colon cancer patients, we detected alterations in positions 450 (c.450_478del29), 455 (c.455delC), 475 (c.475G>A), and 487 (c.487T>A). With an emphasis on some of these mutations, we might mention, for instance, that position 822 (c.822T>A) of exon 8, where we can find synonymous mutations among the samples considered healthy, is near position 818, where alterations occurred in as many as four cancerous samples. 

By inspecting variants described in the TP53 Database (URL: https://p53.fr/tp53-database accessed on 1 October 2021 [9], we can declare that many of the mutations found in the genes of patients with colorectal cancer have been described previously. By examining the variants obtained in exon 5, we can conclude that the c.524G>A (p.Arg175His) variant was also most frequently described in colorectal tumors (15.79%), breast cancer (10.69%), and ovarian tumors (8.80%). This is similar to the c.742C>T (p.Arg248Trp) variant observed in exon 7, which has been described to occur at a greater frequency in colorectal tumors (12.86%) than in other types, such as breast cancer (10.83%) or ovarian tumors (7.85%). Further analysis of the variants obtained in exon 8 revealed that the c.818G>A (p.Arg273His) variant was most frequently described in colorectal tumors (11.66%), but it was also observed in breast cancer (11.07%) and ovarian tumors (10.96%). Similarly, the c.844C>T (p.Arg282Trp) variant was more frequently described in colorectal cancer (14.63%) than in other cancers such as esophageal cancer (10.50%) or brain tumors (8.61%). In conclusion, by analyzing the mutations described in the TP53 Database, it can be stated that the variants most frequently associated with colorectal cancer were predominantly observed in exon 8. Therefore, it can be predicted that the regions corresponding to these mutations represent specific mutational hotspots in colorectal cancer.

## 3. Discussion

The identification of mutation hotspots within the TP53 gene in colorectal cancer patients represents a significant advancement in our understanding of the molecular mechanisms underlying this malignancy. Our study has revealed exons 5 and 8 as critical regions with a high frequency of mutations, aligning with the existing literature, which underscores the importance of these exonic regions in tumorigenesis. Notably, the mutations identified in exons 5 and 7 contribute to the dysfunctional p53 protein, which impairs its ability to regulate cell cycle and apoptosis, thereby facilitating uncontrolled cellular proliferation. This finding is consistent with the established role of the TP53 gene as a tumor suppressor gene, often dubbed the "guardian of the genome" [2].

Furthermore, the differential occurrence of mutations between healthy individuals and colorectal cancer patients highlights the potential of these mutation hotspots as biomarkers for early detection and risk assessment. This is particularly relevant in the context of personalized medicine, where an in-depth understanding of an individual’s specific mutational landscape can guide targeted therapeutic strategies.

Our bioinformatic approach, leveraging self-developed software (version 1), demonstrates the utility of computational tools in genetic research. The ability to rapidly and accurately identify mutation hotspots presents a valuable asset in both research and clinical settings.

However, it is crucial to acknowledge the multifactorial nature of cancer development. While TP53 gene mutations play a pivotal role, other genetic, epigenetic, and environmental factors also contribute to malignancy. Future research should aim to integrate these diverse factors to provide a more comprehensive understanding of colorectal cancer pathogenesis.

While the functional implications of these hotspot mutations were not directly examined in our study, the existing literature provides insights into their potential roles in cancer pathogenesis. For instance, the c.524G>A mutation in exon 5, which we identified as a frequent alteration, has been previously described as a driver mutation in colorectal cancer and Li–Fraumeni syndrome [12,13,14]. In our samples, the most frequent variant in exon 5 was c.404G>T, which is likely pathogenic. Similarly, the c.637C>T mutation in exon 6 has been associated with both colorectal and breast cancers [15,16], highlighting the potential for shared mechanisms across different malignancies. In addition, the alteration c.818G>A in exon 8, which seems to be a pathogenic variant, has also been described in the context of colorectal cancer [17,18]. In addition, in exon 8, there is another frequent alteration, c.844C>T, which also seems to be pathogenic and appears in context with breast and colorectal cancer [15]. The appearance of these mutations in the literature supports the fact that such sequence variations may occur with higher frequencies in the regions we have defined.

Our analysis identified that mutations in exons 5 and 8 are significantly correlated with aggressive tumor behavior, suggesting potential targets for novel therapeutic interventions.

The mutations described in other malignancies comprise, for instance, the alteration c.1024C>T (rs730882029) in exon 10, which has been described in the context of acute myeloid leukemia [19]. Furthermore, the very common mutation c.818G>C (rs28934576), appearing in exon 8, has been observed in breast, pancreatic, prostate, and pulmonary adenocarcinomas, which is also related to Li–Fraumeni syndrome [20,21].

Notably, the observed mutation hotspots align with the findings of other studies, lending credence to our results and underscoring the importance of these mutational events in colorectal cancer development and progression. However, it is essential to acknowledge that the mere presence of mutations in these regions does not necessarily confer a predisposition to cancer, as evidenced by the synonymous mutations identified in the healthy group. This observation reinforces the multifactorial nature of cancer development, where genetic alterations interact with various environmental, epigenetic, and other factors in a complex, multistep process.

These findings are consistent with a previous study by Liu Y et al. (2019) [22], which also identified exon 8 as a hotspot region in advanced lung cancer. However, our study extends previous observations [22,23,24] by highlighting the clinical significance of exon 5 mutations in colorectal cancer. 

A significant limitation of our study was the lack of functional validation or mechanistic insights into the identified mutation hotspots. Although bioinformatics approaches are invaluable for identifying potential gene regions of interest, experimental validation and detailed characterization of the biological consequences of these mutations are crucial for fully elucidating their roles in cancer pathogenesis.

Nonetheless, our findings have potentially valuable implications for cancer diagnosis and personalized medicine. The identification of mutation hotspots could facilitate the development of targeted screening and early detection methods, enabling the identification of individuals at higher risk for colorectal cancer based on their specific mutational profiles. 

The detailed identification and comprehensive analysis of these specific TP53 gene mutation hotspots have the potential to significantly contribute to the advancement of more precise and effective screening tools, as well as the development of targeted therapies tailored to individual genetic profiles. This, in turn, could lead to substantial improvements in patient outcomes and overall treatment efficacy for those suffering from colorectal cancer. By focusing on these mutation hotspots, researchers can enhance their understanding of the disease’s molecular mechanisms, paving the way for innovations that may transform clinical approaches and patient care in this domain.

Future research should focus on functional validation studies to explore the mechanistic underpinnings of the identified hotspot mutations and their impacts on cellular processes such as cell cycle regulation, apoptosis, and DNA repair. In addition, large-scale genomic and transcriptomic analyses could further elucidate the interplay between TP53 gene mutations and other genetic and epigenetic alterations in colorectal cancer, providing a more comprehensive understanding of the complex molecular landscape of this malignancy.

In conclusion, our findings offer promising avenues for the development of enhanced diagnostic and therapeutic strategies by focusing on the identified TP53 gene mutation hotspots.

## 4. Materials and Methods

In our study, we compared the TP53 gene sequences of 50 samples from healthy individuals with those of 50 samples from patients diagnosed with colon cancer. Based on the results obtained, we subsequently identified the most frequent TP53 gene mutation hotspots present in colon cancer. 

The 50 samples from healthy individuals were obtained from the bioinformatics data repository entitled UMD TP53 Database (URL: https://p53.fr/tp53-database, accessed on 1 October 2021), which lists the different variants of the TP53 gene that have already been described and enables their targeted screening [8,9]. The database enables filtering of the variants of the TP53 gene based on user-defined criteria, such as mutation type, exon/intron number, effect of the variant, or the type of nucleotide change. Functional and structural data can be used for filtering, but variants with proven relevance to tumorigenesis can also be searched for. Variants not associated with functional divergence were screened, and the search was adjusted to specifically filter for gene sequences carrying synonymous mutations. Subsequently, 50 variants, corresponding to randomly generated numbers, were selected from the list. Therefore, these samples only contained mutations synonymous with the current GRCh38.p14 primary assembly, meaning that according to the database, no functional alterations already described can be linked to them. Although these 50 healthy samples were selected randomly, we were sure to include at least three variants in the set that contained mutations that were individually synonymous with each exon of the gene. This was important because we aimed to examine the mutations found in samples from patients with colorectal cancer and to compare them with these synonymous mutations, which were considered not associated with functional divergence or cancer development. This could indicate that certain mutations, occurring at specific genomic positions, are more likely to be associated with the pathogenesis of colorectal cancer than others. The samples from colon cancer patients were obtained by courtesy from the Department of Pathology at the Emergency Clinical Hospital Targu-Mures, Romania, and were granted permission to be used in this study by the Ethics Committee. These samples were collected in 2017 from patients treated at the hospital, and given the relatively challenging and costly sequencing of the TP53 gene, they were sequenced at Hamamatsu University in Japan [23,24]. In total, we gathered 120 samples (i.e., 60 healthy samples and 60 cancerous samples), of which we were able to assemble a representative sample according to the proportions of the total number of samples that contained sequences with detectable mutations in the gene structure, and a few had no structural changes that could be observed regardless of the presence of colon cancer. 

**Figure 4:**
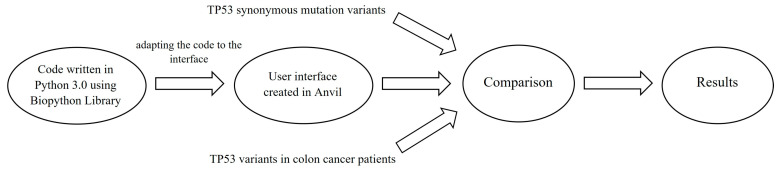
A workflow diagram of the self-developed computer program. We first wrote the algorithm in Python 3.0 using BioPython libraries and created a user interface for it in Anvil. In its application, TP53 gene sequences of patients diagnosed with colorectal cancer were introduced, as well as those of healthy individuals containing synonymous mutations. These variants were then compared exon-by-exon, resulting in sequence alignments.

Our samples were processed based on repeated comparisons that were made possible using self-developed software (version 1). We compared these samples for each exon, particularly for attaining greater clarity and transparency. The software was written in Python 3.0, which is based on the BioPython library that allows sequence alignment and repeated comparison, which can determine the exact degree of similarity. Python was used because it is an object-oriented, flexible programming language that is particularly suited to the specific challenges posed by scientific questions of interest. Therefore, it is widely used for carrying out this type of computation. The BioPython library is a collection of freely available Python modules for computational molecular biology, providing a wide range of Python-based resources for bioinformatics use and research [10]. For our purposes, we used a pairwise sequence alignment algorithm from the BioPython library. This algorithm performs pairwise sequence alignment, which is the process of aligning two sequences by optimizing the similarity score between them. The BioPython library contains a Bio.Align module, which can perform this task with the Pairwise Aligner class using global and local pairwise alignment algorithms. The program reads the given exon’s sequences into a variable and then compares them using the above-mentioned sequence alignment algorithm. The result of this alignment is initially printed, giving the option of downloading it or storing it in a .txt document. To provide the correct input for the program, namely the gene sequences to be compared, we needed an interface that would allow us to do this in a more visually intuitive and user-friendly way. To do this, we used an online application program called Anvil, which provides a good alternative for creating an online interface for algorithms running in Python in a relatively simple manner (Figure 4). With the help of this program, computer codes running in the background also receive a front-end user interface, the language of which is English, to ensure broad access. In this way, we can specify in detail how certain elements should look like on the screen and, if we click on one of them, which part of the algorithm should be accommodated. Each element has a specific function and trigger, which determines when that element is activated. With this, we were able to create a button labeled UPLOAD, which allows us to select the gene sequences to be compared. This acts as a file loader through which we can provide inputs to the algorithm. In Anvil, we have created a button labeled COMPARE, which, when triggered, will start the comparison, and display the result. This is also an action button that performs a user-specified task, and when we press it, the result of the given comparison is displayed. In addition, it is possible to download the obtained result by pressing the button labeled DOWNLOAD, in which case a given sequence alignment is saved into a .txt file, which can then be downloaded to the user’s computer. This process can be repeated sequentially, allowing for all possible gene sequence comparisons.

The source code is deposited at our Oncopathology and Translational Medicine Research Centre and can be accessed upon request, and T.A. Gyorgy is responsible for responding to the request for computer code.

**Figure 5:**
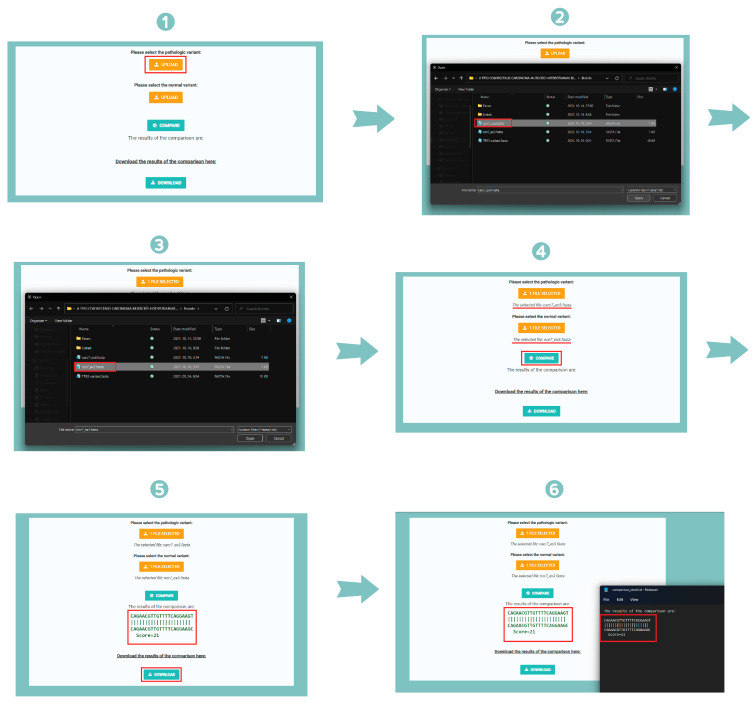
The operation process of the self-developed computer program. First, we selected the DNA sequence for the pathological variant from a colon cancer patient by clicking on the button labeled UPLOAD. After this, we inputted the DNA sequence for the variant from a healthy individual by clicking on the second button labeled UPLOAD. Then, we clicked on the button labeled COMPARE, to output the juxtaposition of the two DNA sequences inputted, and a sequence comparison score was computed and displayed. To download the results of the comparison, we clicked on the button labeled DOWNLOAD.

The self-developed computer program is quite easy to use, as illustrated in Figure 5. Here, we compared a variant of exon 3 from a healthy sample with one from a sample with colon cancer. To begin, we click on the first button labeled UPLOAD, and then in the pop-up window, we select the sequence for the pathological variant stored in .FASTA format. We used this file extension because bioinformatics databases regard this file format as one of the fundamental file formats for expressing various types of biological sequences; however, the computer program will accept the .txt format as well. After inputting the DNA sequence containing the pathological variant to be compared, the selected filename will be shown immediately, allowing us to move on to input the DNA sequence containing the variant from a healthy individual by clicking on the second button, labeled UPLOAD, and then the .FASTA file containing this sequence is displayed in the pop-up window. The filename will be shown immediately this time as well, which tells us that the program is ready to perform the comparison, so that we can click on the button labeled COMPARE. As a result, the computer program will output the juxtaposition of the two DNA sequences that are inputted and will also display a sequence comparison score. This score is calculated by a system based on a specific formula in which, in addition to identical nucleotides, substitutions and gaps also play an important role. This pairwise score is calculated for each pair of sequences to be aligned and is based on the number of identical residues contained in the optimal alignment divided by the number of aligned residues compared. In this study, we only aimed to detect the mutation positions by comparing the gene sequences of patients with colorectal cancer to those of healthy individuals; we did not take into consideration the sequence comparison score of pairwise sequence alignments.

If we wish to store the results obtained, we may do so by clicking on the button labeled DOWNLOAD, in which case the program will download the results of the comparison for us stored in a. txt document. After this, we may start the process again, performing a comparison on another pair of exons.

## 5. Conclusions

Comparing the TP53 gene sequences of the samples from healthy individuals containing synonymous mutations to those of the samples from patients diagnosed with colon cancer led us to conclude that the mutations identified in them are in close proximity to each other, often only a few positions apart from one another. The fact that structural alterations occurring in similar regions may in certain cases be associated with an increase in the susceptibility to malignancies, while in other cases, they may result in function-preserving mutations that have no substantial impacts on a physiologically healthy state, which supports the assumption that the development of cancerous cell proliferation hinges on the combined presence of several factors. Therefore, the pathological mechanism of cancer is multifactorial.

## Data Availability

The original contributions presented in the study are included in the article, further inquiries can be directed to the corresponding author.
